# Designing Men’s Health Programs: The 5C Framework

**DOI:** 10.1177/15579883231186463

**Published:** 2023-07-26

**Authors:** Paul M. Galdas, Zac E. Seidler, John L. Oliffe

**Affiliations:** 1 Department of Health Sciences, University of York, York, UK; 2 Orygen, Melbourne, Victoria, Australia; 3 Centre for Youth Mental Health, The University of Melbourne, Melbourne, Victoria, Australia; 4 Movember, Melbourne, Victoria, Australia; 5 School of Nursing, The University of British Columbia, Vancouver, British Columbia, Canada; 6 Department of Nursing, University of Melbourne, Parkville, Victoria, Australia

**Keywords:** men’s health, masculinity, gender-transformative, help-seeking, men’s health programs

## Abstract

Men are less likely than women to access or engage with a range of generic health programs across a diversity of settings. Designing health programs that mitigate barriers associated with normative ideals of masculinity has been widely viewed as a key factor in how health systems should respond, but strategies to engage men have often narrowly conceptualized male health behavior and risk inadvertently reinforcing negative and outdated gender stereotypes. Currently absent from the men’s health literature is practical guidance on gender-transformative approaches to men’s health program design—those which seek to quell harmful gender norms and purposefully promote health equity across wide-ranging issues, intervention types, and service contexts. In this article, we propose a novel conceptual model underpinned by gender-transformative goals to help guide researchers and practitioners tailor men’s health programs to improve accessibility and engagement. The “5C framework” offers key considerations and guiding principles on the application of masculinities in program design irrespective of intervention type or service context. By detailing five salient phases of program development, the framework is intended as a designate approach to the design of accessible and engaging men’s health programs that will foster progressive changes in the ways in which masculinity can be interpreted and expressed as a means to achieve health for all.

## Introduction

It is widely recognized that men are less likely than women to access (i.e., seek, utilize) or engage with (i.e., have active and/or sustained involvement) a range of generic health programs across a diversity of settings; irrespective of age, nationality, or ethnocultural background (Sharp et al., 2022). Gender-specific barriers and stigmas for men’s help-seeking associated with conformity to culturally normative ideals of masculinity have been reported as contributing factors to many men’s reticence to utilize health services. For example, tolerating pain and delaying visiting the doctor for minor problems has been argued to reflect idealized (Western hegemonic) masculine constructs of “hardiness” (O’Brien et al., 2005). Men’s resistance to seek help for psychological problems have similarly been attributed to normative masculinities characterized by self-reliance, stoicism, and restrictive emotionality (Seidler et al., 2016). More broadly, research finds men who adhere to dominant ideals of masculinity experience worse mental health outcomes, engage in more risk-taking health behaviors (e.g., smoking, excessive use of alcohol), and use violence to demonstrate power more than men who challenge dominant notions of masculinity (Dworkin et al., 2015; World Health Organization, Regional Committee for Europe, 2018a).

The design of health programs and services and the manner in which they are delivered are key factors influencing the way health systems around the world respond to men’s health needs (World Health Organization, Regional Committee for Europe, 2018b). As evidence of men’s gender-related constraints to access and engagement has developed, gender-sensitization emerged as best practice for designing tailored health programs (Fleming et al., 2014). Reflecting a shift from generic “gender-neutral” approaches that do not account for the gendered contexts that shape health, gender-sensitive health programs are defined as those which recognize the differential needs (and constraints) of men and women (Barker et al., 2007; Fleming et al., 2014; Gupta, 2000). There is considerable evidence that gender-sensitized models of health care and service delivery which incorporate understandings of masculinities by aligning with the specific needs, concerns, preferences, and capacities of men can yield benefits in uptake and engagement (Sharp et al., 2022; Struik et al., 2019; World Health Organization, Regional Committee for Europe, 2018b). Examples include community-based health promotion initiatives (Bergin & Richardson, 2021), mental health services (Seidler et al., 2016; Seidler, Rice, River, et al., 2018), physical activity (Seaton et al., 2021), weight-loss programs (Hunt et al., 2020), and self-management support interventions for chronic illness (Galdas et al., 2014).

**Figure 1.**
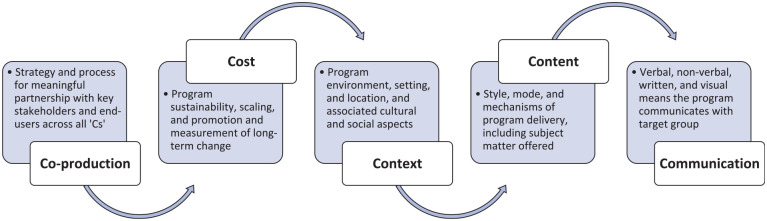
The 5C Framework

Underpinned by this evidence-base, broader recommendations for integrating gender-related influences in the design, planning, implementation, and evaluation of health programs for men have emerged. While advancing the field, existing guidelines are limited to those with a clinically specific focus (e.g., mental health/psychological treatment Seidler et al., 2016), particular population (e.g., fathers King et al., 2004), or intervention type (e.g., health promotion programs Struik et al., 2019). Conspicuously absent from the men’s health literature is a designate approach to integrating masculinities into program design across diverse service contexts and settings. In this article, we propose a novel conceptual model that seeks to address this gap. Informed by key literature and our long-standing work in the field, the “5C framework” (see Figure 1) is intended as a practical tool to guide researchers and practitioners aiming to tailor new or existing health programs to improve accessibility, engagement, and appropriateness for men. By detailing five salient phases of program development relevant to the impact of gender and the intersections with other social determinants of health, the framework offers key considerations and guiding principles on the application of masculinities irrespective of intervention type, service context, or clinical focus.

## A Gender-Transformative Framework

A preponderance of approaches to gender-sensitization has involved “male-friendly” programs designed to mitigate barriers to access and engagement associated with normative ideals of masculinity. Though well-intentioned and often effective at “getting men through the door” in the short term, the strategy risks being complicit in reinforcing the unhelpful gender norms that these interventions are seeking to address; a counterproductive state to achieving sustainable health outcomes and gender equality (Fleming et al., 2014). Examples of this tactic range from housing health interventions in local pubs and bars to improve recruitment of men, to language employed leaning on masculine tropes of bravery and courage when it comes to speaking up and seeking help. Gender-transformative approaches, by contrast, have garnered growing attention as a means for *reshaping* gender relations toward equity by actively challenging prevailing gender stereotypes and offering positive alternatives to effect lasting change in gender norms and health outcomes (Dworkin et al., 2015). Researchers have argued for greater awareness of ways to attend to the plurality of masculinities, and the broader social contexts within which masculinities are defined and produced in an effort to achieve gender-transformative health programming with men (Dworkin et al., 2020; Evans et al., 2011; Seidler, Rice, River, et al., 2018). Our 5C framework builds on this work, moving from a gender-sensitive orientation that narrowly conceptualizes men’s concerns and behaviors, toward a gender-transformative approach predicated on challenging (and changing) unhelpful gender norms with the goal of making accessible and engaging health programs and services normative within an inclusive masculinities context (Anderson & McCormack, 2018; McGrath et al., 2022; Seidler, Rice, River, et al., 2018). Our aim is to guide the development of care and service delivery that improves men’s uptake and engagement across the life-course by transforming hegemonic ideals, fostering gender equity, and democratizing men’s relationships (Fleming et al., 2014; World Health Organization, Regional Committee for Europe, 2018a).

## The 5C Framework

### Co-Production

The initial concept in the framework, *co-production*, refers to the identification of a strategy and process for meaningful partnership with key stakeholders and end-users, and this should be viewed as an overarching principle and permeating activity for the other four “C”s. Approaches to co-production in health care services and research (e.g., co-design; co-creation; co-implementation) have been widely reported and can involve stakeholder and public engagement through involvement in any or all steps of service design, delivery, and evaluation. We have found that engaging stakeholders as early as possible and agreeing on a strategy for co-production and the principles that will guide its implementation is a critical antecedent to well-designed programs that are effective in engaging men.

The way co-production is enacted and operationalized varies depending on its end goal. Key to success in gender-transformative co-production is consideration of the diversity of men’s needs, which are likely to reflect the disparate patterns of masculinities that stem from intersections between gender and other social determinants of health (Evans et al., 2011). Help-seeking behavior patterns and service needs differ among men of different age, socio-economic background, sexuality, or ethnicities. There is unlikely to be a “one-size-fits-all” model of service delivery that improves program accessibility and engagement among all men in all contexts and historically, men’s uptake in such exercises is led by existent proactive help-seekers. Program development should begin with the collaborative identification and purposeful seeking out of a target population and/or subgroups of men who may be most disadvantaged and at risk of the health issue(s) of relevance. Experience has taught us that reaching beyond the individual level to adopt a “whole systems” approach to co-production—for example, partnering with workplaces, social institutions, religious, or community leaders—can afford opportunities to garner critical insights into structural and social determinants of health service utilization that may be patterned within subgroups of men (Baker, 2018; Fisher & Makleff, 2022). This multipronged recruitment strategy can help reduce self-selection bias and aid the scaling of gender-based health interventions and programs, which necessitates whole system partnership in the process of program evaluation and dissemination (see “Cost,” below). For example, central to the successful development, delivery, and subsequent scaling of the Football Fans in Training (FFIT) men’s weight management program was a whole system co-production strategy which included male end-users, health professionals, fitness coaches, and the Scottish Premier League (SPL) Trust which had the remit to deliver social change through community engagement within professional football clubs in Scotland (Hunt et al., 2013).

Co-production that aims to inform the development of programs targeting men should therefore extend further than consulting individuals about their views and preferences. It requires a reciprocal partnership in the processes of design, delivery, and evaluation that facilitates consideration of the ways in which diverse groups of men can be supported to feel it is acceptable to seek help and express themselves in healthy ways within the “context” (see below) a program is being delivered (World Health Organization, Regional Committee for Europe, 2018a). When selecting approaches to support the achievement of these goals, it is important that they are defined early in the planning process, sustainably resourced, and clearly aligned with the overall co-production strategy and desired outcomes (e.g., defining program outcomes; gathering insight into health needs, cultural beliefs, and gender norms; use of language; program content; environmental and structural considerations). A range of approaches may be needed to tailor co-production to diverse contexts, stakeholder groups, and stage of program development. Informal activities have shown particular promise as a means of achieving gender-transformative outcomes for men, recently exemplified in the development of the “Sheds for Life” program (McGrath et al., 2022). An informal approach to co-design workshops facilitated normed, meaningful conversations, openness about vulnerability, and the broaching and reframing of ordinarily taboo health topics such as depression. Men were noted to progress from a belief that discussing health was “*not what men do*” or a “*failing*” to consideration of what it meant to be a man and engage self-health in a more meaningful way (McGrath et al., 2022). We have found that combining informal approaches with more structured deliberative methods such as nominal group technique and the Delphi method is effective (Cantrill et al., 1996), particularly when the identification of priorities or formation of consensus is a key objective. For example, the BALM study (www.balmprogramme.co.uk) utilized consensus group methodology involving a diverse group of men working in frontline health care roles to co-produce a guided self-help mental health intervention. Groups comprised men across a range of age, ethnicity, and job role, including trade union and occupational health representatives. Consensus group workshops were oriented around different aspects of intervention design and delivery (e.g., content, language, support mechanisms, and structure) that would appeal to men and help overcome gender and occupation norms seen to act as a barrier to accessing early mental health support.

### Cost

We define “cost” as relating to matters of program sustainability, scaling, and promotion and measurement of long-term change. Budget implications for men’s health programs are ever-present and, related to co-production, we recommend developers initiate formal discussions early on with partners to decide who pays for what in the start-up phase. This should extend to definitive plans for sustaining (and where appropriate scaling) that investment beyond the launch and pilot testing phases. The cyclic nature of research grants, government budgets, and nonprofit investments routinely seed men’s health programs, but those catalytic funding models and mechanisms significantly challenge sustained delivery. The tradition in public health is that health care is free, and we have found that these traditions can limit the feasibility for recouping or covering program costs via end-user payments. Moreover, targeting men experiencing health inequities is at odds with charging individuals from such subgroups to access the tailored programs we design and espouse as freely needing. Instead, programs most often compete for men’s time and thus, their costs for participation are typically indirect. Programs therefore need to be asset-building (i.e., focus on developing protective factors and resources for embracing positive and health-conscious aspects of masculinity) to justify their time involvement. A related conundrum here is that program leaders and settings/environments should be compensated for retention, and to gauge the true program costs. Lacking these initial and ongoing cost considerations, experience has taught us an unfortunate truth that many men’s health programs rely on (and die with) one-time funding, with some lingering based only on the good-will of individuals and potpourri fundraising. Rigorous evaluation is essential, which should be built-in from the outset, planning-specific data collection and analyses that match the program’s development stage (Oliffe et al., 2020). Evaluation should reflect the intended intervention purpose and outcome measures of importance to end-users, as well as economic considerations (e.g., cost benefit or cost consequence analysis) that can provide potential funders with a comprehensive guide as to the likely cost-effectiveness of programs. This has informed the strategic funding intentions of Movember, the leading men’s health charity globally, where seed funding across multiple countries has primed a range of grassroots health service projects that have shown promise, but often lacked the plans and actions necessary to fully establish innovation in policies, programs and service delivery. Focus has therefore turned to directing funding to strengthen the implementation and evaluation of potentially scalable initiatives to ensure they get into an implementation and/or partnerships play.

In securing sustainable funding pathways, important lessons can be gleaned from the commercial determinants of health, who have long-standing track-records in using effective strategies for gendered marketing and selling to men to produce sustainable funding streams. Although alluring, we advise caution in the use of strategic promotions and commercial partnerships which trade longer term gender-transformative goals for short term gains in service user engagement or sustainable financing (see “Communication,” below). In some instances, commercial strategies are somewhat pernicious, such as in the norming (i.e., selling) of gambling and alcohol within corporate sports and events. Such approaches leverage harmful gender norms (e.g., alcohol consumption as a key practice of hegemonic masculinity) with men’s belonginess to specific teams and leagues, with the promise of predictive expertise and access to associated (and celebratory) substances cementing those allegiances. A less-troubling example is the selling of branded athleisurewear with which men associate health and healthy behaviors (e.g., Nike—Just do it; Under Armor), and program developers might usefully lift some of these marketing elements to help. For example, the *Dads in Gear* program (Bottorff et al., 2017) aimed at fathers reducing and quitting smoking provided branded program caps and t-shirts to forge group identity and equip participants to take part in the physical activity session in the gym where they met each week. With regard to securing ongoing budgets, we suggest one potential avenue is to go direct to corporate sellers of men’s health early in the program development phase—ideally including them as a partner from the outset. An example of this is the *Dad’s Central* program (https://dadcentral.ca/resource-store/), which is in part sponsored by Dove Men, and independently sells merchandise and resources. A range of cause-driven opportunities to engage philanthropists and investors who share program values and long-term aims maybe available, but we strongly recommend these business case efforts should be built from the outset as partnerships.

### Context

“Context” refers to the features of a men’s health program’s environment, setting, and location and its associated cultural and social aspects (Craig et al., 2018). Understanding how these contextual factors shape norms, definitions, and practices of masculinity among the target population or within subgroups of men, and how they can be leveraged to encourage positive healthy masculinities that norm health program access and engagement, is vital to gender-transformative service design and delivery.

Decisions around intervention context should be grounded in an understanding that masculinities and men’s health practices reflect a patterned set of social interactions influenced by and situated within institutions such as workplaces, community organizations, sports clubs and teams, social venues, and so on, which can be regionally and nationally determined (Dworkin et al., 2015). As we have discussed, co-production activities are a crucial initial step in garnering expert knowledge and lived experience on the context within which a program can be delivered. These understandings should then inform decisions related to every aspect of program design to maximize access and engagement in the target population (Oliffe et al., 2020).

Research has highlighted the value of anchoring service delivery within contexts where men live, work, and socialize, to enable men to connect with others who experience and manage similar or relatable challenges within a shared sociocultural context (Sharp et al., 2022). Community-based programs delivered in men-friendly spaces now have a substantial evidence base as providing a familiar and acceptable environment that can promote men’s access and engagement. This has been reported to be particularly relevant for engaging marginalized subgroups of men who may have a distrust of, and greater reticence to engagement with, traditional health care environments (Oliffe et al., 2020).

The use of professional sports clubs (e.g., soccer, rugby, ice hockey) has been the prevailing model of aligning program context with the identities of a male target population. Success in seminal men’s health promotion programs Football Fans in Training (UK) (Hunt et al., 2013) and HAT-TRICK (Canada) (Caperchione et al., 2021) were founded on collaborative partnerships with community-based organizations and teams that reflect local and regional cultures and interests (soccer and ice hockey, respectively). A common theme running through these programs is the benefits afforded by the deep social and cultural ties men have to sporting contexts, which reflect nationally and regionally situated intersections of culture and gender. Of key importance more broadly is the delivery of service within a familiar context that can leverage placed-based normative masculinities to increase the relatability of health interventions and improve men’s access and engagement as a result. This principle can extend to a broad array of traditional and nontraditional masculine settings but requires careful consideration to avoid inadvertently strengthening adherence to hypermasculine environments, such as sports clubs. To avert this pitfall, we advise considering context as a way of providing a “safe and courageous space” to work *with* as well as *rework* masculine ideals associated with men’s engagement in health services in specific locales (Sharp et al., 2022). When designed appropriately, intervention context can then afford men a greater awareness of normative attitudes in a setting or community and facilitate critical discussions, questions, and transformation of these norms (Barker et al., 2007). For example, participants in the HAT-TRICK program were reported as embracing masculine values of strength, resilience, and independence to make positive health behaviors contextually relevant. By inclusively working *with* some social constructs of masculinity, men can align to and argue against dominant masculine ideals embodying an array of configurations within a particular context (Sharp et al., 2022). In this regard, research has demonstrated the importance of weaving contextual and content design considerations that reflect a plurality of masculinities, particularly where aspects of identity such as sexual orientation may be hidden within environments such as sports clubs that are associated with restrictive hegemonic masculinities (McGrath et al., 2022).

### Content

A key decision for developers of men’s health programs involves the selection of intervention “content” that not only yields positive health outcomes but is also appealing and engaging to the target group. We define “content” as the style, mode, and mechanisms of program delivery, as well as the subject matter being offered. Successful programs are typically complex, involving multiple interacting content components, and recommendations on what “active ingredients” work to improve acceptability and accessibility for men are diverse.

Consistently highlighted across a range of service evaluations is the value of content that is based on activities familiar and appealing to men. Interventions framed around men’s skills, interests, and hobbies have garnered particular attention as a way to play to diverse masculine ideals such as independence, self-reliance, and problem-solving. Differing in mode (e.g., online, in person, group-based) and range of activity (e.g., woodwork, music, sport, cooking, gaming), such programs have commonality in offering enjoyment and familiarity in purpose and structure, often with the goal of harnessing social connectedness and/or engaging men directly with health promotion and illness management strategies (McGrath et al., 2022; Oliffe et al., 2020). Other noteworthy content characteristics associated with successful male engagement include an informal style of delivery facilitated by professionals or specially trained peers or role models, which can create opportunities for casual, “shoulder-to-shoulder” learning and peer support as a by-product of the shared activity.

We urge vigilance when considering the deployment of “male-friendly” activity content to avoid prioritizing and reinforcing normative masculinities over more diverse expressions. Key components of gender-transformative programs with men should prioritize content that allows the examining of the role of power relations in negatively shaping health, identifying attitudes and practices among men that harm both women’s and men’s health, and viewing men as active agents of change in advancing gender equity (Dworkin et al., 2020). Programs leveraging normative ideals (e.g., strength, willpower, provider) may successfully “engage men” but may not allow for the challenging of harmful gender norms or unequal power relations. For example, many programs designed to engage fathers and husbands in maternal and perinatal health care have appeared superficially positive, but may inadvertently cause power imbalance and gender disparities when the focus of program content leads men to assume the stance of “protecting” and “looking after” women (Raghavan et al., 2022).

Akin to contextual design considerations, key to success is developing program content as a “hook” that offers participants assets and the permission to critically reflect on masculinity and gender norms in and around the “doing” of an engaging activity (Caperchione et al., 2021; Oliffe et al., 2020). Those delivering or facilitating programs may usefully achieve this by drawing on personal reflections or through participatory content such as role-playing, case studies, or “what-if” activity based scenarios (Barker et al., 2007). For example, men attending the Men’s Health and Wellbeing Program (MHWP) in Ireland involving information sessions, cookery classes, and health checks by nurses, recognized how male instructors and session facilitators actively challenged traditional gender norms or stereotypes through their own practice or lived experience, such as staying away from the kitchen, abstaining from conversation, or avoiding collaboration/teamwork (Lefkowich et al., 2017). Men suggested that the program had an additional “ripple effect” that increased their capacity to confront gender norms and take on more active roles at home and in the community.

Indeed, emerging evidence indicates gender-transformative programs are multilevel; drawing on strategies that reach beyond target groups to mobilize the wider community to adopt egalitarian gender norms and practices (Ruane-McAteer et al., 2020). In practice, this means developers considering a range of content that will help work toward structural and community-level changes in behaviors and attitudes, such as institutional policies specific to the context in which individual choices are enacted (e.g., workplaces), rather than solely focusing on the individual (Dworkin et al., 2015). Drawing again from the lessons of fatherhood programs, targeting health provider policy to enhance men’s engagement in routine maternal health care has been noted to achieve sustained change promoting gender equality (e.g., protecting women, families and children from violence), and not solely activities that focus on individual fathers’ attitudes toward gender norms and fatherhood (Raghavan et al., 2022). Mechanisms for supporting structural and community-level change are nascent and we echo calls for developers to implement rigorous evaluations of gender-transformative programs to help generate evidence to guide the future work of others on ways to scale up promising approaches (Dworkin et al., 2015).

### Communication

The final concept in the 5C framework, “communication,” relates to the verbal, nonverbal, written, and visual approach with which a men’s health program communicates with its target group. Understanding and working with men’s preferred language and style of communication can have a substantial influence on uptake and engagement and is widely recognized as an important tool in the provision of a male-centered approaches. Key dimensions to consider include ensuring program labeling, marketing, and promotion is understandable, accessible, and inclusive of men from diverse backgrounds, and adopting a style and expression during the process of content delivery that aligns with the target group’s language preferences and health literacy (Oliffe et al., 2020). There is broad consensus in the literature around a number of strategies of potential benefit in this regard. These include adopting a non-jargonistic conversational approach; being frank, direct and honest; use of lay and colloquial language and terms; using labels and metaphors that connect with men’s interests (e.g., related to sports, building, fixing, or computing); and the appropriate use of humor (Seidler, Rice, Ogrodniczuk, et al., 2018). Some studies have recognized that allowing silences can be an important feature of communication strategies, aiding men’s focus for an activity-based content and/or allowing time to think and reflect independently (Oliffe et al., 2020).

There is a particularly strong evidence-base for these communication considerations in psychological health programs, where research has consistently identified that medicalized language, jargon or labels using “mental health” or associated terms can be barriers to engaging men. Here, working with men’s language preferences, and avoiding pathologizing medical terms in particular, has been demonstrated to assist in attendance (Oliffe et al., 2020). The Canadian Veteran Transition Program offers an illustrative example. A group-based mental health service for men returning to civilian life after the military, the program did not employ medical language and was not framed as “counseling” or “mental health,” but instead drew on colloquial language to engage men in the work of “dropping their baggage” (Kivari et al., 2018). These principles can extend more broadly across a range of service and clinical foci to help reduce stigma and aid health literacy, such as in the use of proverbs, songs, stories, games, images, and metaphors to convey messages in a relatable manner.

That said, congruent with the aforementioned challenges that run through the previous “C”s,’ it is not sufficient to deploy language or images that are relevant and appealing to men without considering the ways in which they may reify harmful aspects of masculinity that programs should be working to change. Fleming et al. (2014) critical assessment of the “Man Up Monday” public health campaign in the United States presents a powerful case in point. Here, savvy advertising using images and language that included a photo of a bed, boxer shorts with a fishhook inside them, and taglines such as “*If you hit it this weekend, hit the clinic Monday”* were deployed to encourage men to get tested for sexually transmitted infections (STIs). Resulting in a 200% increase in the number of men that tested for STIs, the program clearly resonated with some men. Yet, drawing on colloquial calls to *“man up,”* and using imagery (a bed, underwear) and phrases such as “*if you hit it*” (referring to sex) norming narrow constructions of manhood based on sexual prowess and conquest, are known to reinforce harmful health outcomes in the longer term (Fleming et al., 2014).

Program developers aiming to adopt gender-transformative communication strategies thus need to resist the appeal of patriarchal parody for short term gains in male uptake, and instead consider communication strategies that embody healthy masculine states and relations. Recommended in this example was a messaging strategy examining the norms that lead men to risk unprotected sex and have multiple partners to take preventive action to decrease the likelihood of contracting (or spreading) an STI (Fleming et al., 2014). To avoid stereotypes and resist communication based on gender inequities, we encourage program developers to switch the way they look at language and imagery to help reveal where power and control exist. For instance, in the “Man Up Monday” example, considering a program involving images portraying female underwear with a fishhook inside and asking women to get tested “*if you hit it*” at the weekend can help prompt reflection on unequal power structures and gender dynamics inherent in the messaging being deployed (Affiat et al., 2022).

## Conclusion

Guidance has hitherto been lacking on how masculinities can be integrated into the design of men’s health programs using a gender-transformative approach across a diversity of intervention types and service contexts. In this article, we have proposed a novel conceptual model that offers key considerations and principles to help guide researchers and practitioners aiming to develop new or tailor existing health programs for men. The “5C” framework builds on existing guidelines, checklists, and recommendations (Evans et al., 2011; King et al., 2004; Seidler, Rice, River, et al., 2018; Struik et al., 2019) by offering guiding principles in five phases of program design that can be used as reference point for developers to practically consider. Underpinned by gender-transformative goals, a consistent message throughout our guidance has been the importance of thoughtful development to avoid the pitfalls of reinforcing negative (and outdated) gender stereotypes, with the overall goal of program design that tilts toward mechanisms which directly re-address the norms of masculinity that harm health and promote positive health changes for women and men.

Developers seeking to leverage understandings of masculinities in the design of health programs will seldom be able to mitigate the risks of all potential harm. However, as Robertson et al. (2018) have discussed, a balance can be struck in engaging men in ways that utilize aspects of masculinity to improve uptake and engagement, without simultaneously (or inadvertently) reinforcing negative health practices. Application of the 5C framework offers avenues for striking this balance, leading to program designs that foster progressive changes for the ways in which masculinity can be expressed as a means to achieving health for all.
